# Real-World Outcomes of Incurable Cancer Patients Treated with Unlisted Anticancer Treatments in an Academic Center in Quebec, Canada

**DOI:** 10.3390/curroncol31100440

**Published:** 2024-10-01

**Authors:** Adam Miller, Francois Panet, Victoria Korsos, Wilson H. Miller, Gerald Batist

**Affiliations:** 1 Department of Medical Oncology, Queen’s University, Kingston, ON K7L 3N6, Canada; adam.miller@medportal.ca; 2 Jewish General Hospital Segal Cancer Center, Montreal, QC H3T 1E2, Canada; francois.panet@mail.mcgill.ca (F.P.);; 3 Departments of Medicine and Oncology, McGill University, Montreal, QC H3A 0G4, Canada; 4 Department of Hematology, Jewish General Hospital, Montreal, QC H3T 1E2, Canada; victoria.korsos@mail.mcgill.ca

**Keywords:** Quebec, Canadian Agency for Drugs and Technologies in Health (CADTH), Institut national d’excellence en santé et services sociaux (INESSS), incurable cancer, unlisted medications, drug approval

## Abstract

Medical oncology is a rapidly evolving field, with new medications being discovered yearly, contributing to increased survival rates. However, accessing drugs in a timely manner can be challenging. In Quebec, Canada, a physician can prescribe an unlisted anticancer treatment through a regulated pathway under exceptional circumstances. We conducted a quality improvement study describing the outcomes of incurable cancer patients receiving unlisted anticancer therapy at the Jewish General Hospital between 2018 and 2019. Though our study did not include a comparator arm, unlisted anticancer therapies were associated with interesting median progression-free survival (11 months) and overall survival (25 months). Moreover, a large proportion of treatments, 44%, were subsequently reimbursed in the province of Quebec. Given the delay in anticancer drug reimbursement, this pathway is essential for timely access to oncology drugs. Such ‘special access’ programs will likely become increasingly important as precision medicine becomes the standard of practice.

## 1. Introduction

Medical Oncology has been an area of significant development. From 2010 to 2019, it accounted for over 25% of all new drugs approved by the Food and Drug Administration (FDA) in the United States [1]. New anticancer medications have also improved patients’ outcomes [2,3]. In Canada, once Health Canada approves a medication based on its therapeutic quality, there is a cost-analysis evaluation and reimbursement recommendation by Canada’s Drug Agency (CDA), previously known as Canadian Agency for Drugs and Technologies in Health (CADTH). Quebec is the only province with its own governing body that makes recommendations on reimbursement, the Institut national d’excellence en santé et services sociaux (INESSS). Afterward, there is a price negotiation at the pan-Canadian Pharmaceutical Alliance (pCPA) [4]. Finally, based on the recommendations of INESSS, the Minister of Health and Social Services is responsible for deciding to add a medication to the reimbursement list for Quebec [5]. As healthcare is a provincial responsibility, each province decides on its reimbursement policy [4]. This sequential multi-step process delays access to new anticancer medications in Canada, and is potentially detrimental to patients’ outcomes [6,7]. For example, a recent report analyzed the delay from the time of Health Canada approval until reimbursement, revealing a median delay of 379 days for 43 oncology drugs [8].

The delay in the approval of new anticancer medications in public healthcare settings can impact patients’ outcomes negatively [9,10]. In Canada, the delay in approval and reimbursement of three anticancer drugs in non-small cell lung cancer (NSCLC), nivolumab, afatinib, and pemetrexed, was estimated to have affected 6400 patients who lost 1740 person-years [9]. Incurable cancer patients often cannot wait to access new anticancer medication, and the approval process is often too slow, even after Health Canada’s approval [8].

In Quebec, physicians who want to prescribe a medication that is not on the provincial reimbursement list must apply via a mechanism called “formulaire de demande d’utilisation d’un médicament pour des motifs de nécessité médicale particulière”, which is a request form for the use of a medication for reasons of special medical necessity. While there is some variation between institutions regarding how the request is approved, the general rules are that this request must be discussed with other physicians and be approved by a council of doctors, dentists, and pharmacists from the hospital, known as the Conseil des médecins, dentistes et pharmaciens (CMDP). The drug is then paid for via the hospital pharmacy budget or an access program from the drug manufacturer. At the Jewish General Hospital, the drug request must be supported by published evidence and approved by many experts, including the head of the department and at least three physicians. This usually is part of the function of tumor-specific multidisciplinary committees. In this article, we will use the term “unlisted” to describe the drugs accessed by this method.

We conducted a retrospective quality improvement study at a single center to assess the real-world outcomes of patients with incurable cancer treated with an unlisted anticancer drug. Our secondary objectives were to evaluate the supporting evidence for the treatment requests, describe our patients’ outcomes using this drug access mechanism with those reported in the literature, and determine if the medication was eventually reimbursed for use in Quebec. Together, these assessments provide insights into the efficacy and justification of unlisted treatments, help align real-world practices with clinical evidence, and inform policy decisions regarding medication reimbursement in the province of Quebec.

## 2. Materials and Methods

### 2.1. Study Design

We conducted a retrospective observational study of oncology patients over the age of 18 who received an unlisted anticancer drug for incurable cancer from 1 January 2018 to 31 December 2019. The data for patients who were approved for an unlisted medication were retrieved from a database in the Jewish General Hospital oncology pharmacy in Montreal, Quebec, Canada, via a chart review of electronic medical records, which was completed in December 2023. Patients were excluded from the dataset if they accessed the medication via a clinical trial.

### 2.2. Collected Data

Independent variables from patients were the age, sex, and type of malignancy according to the International Classification of Diseases for Oncology [11]. The type of malignancy was first classified between hematological or solid tumors and then divided into specific organs or types of malignancy. Independent variables related to the pharmacologic treatment were the type of treatment (targeted therapy, immune checkpoint inhibitor and chemotherapy), drug name, line of treatment in the incurable setting, if treatment was subsequently reimbursed in the province of Quebec, and the level of evidence supporting the request (phase III clinical trial or others). The authors retrospectively identified the type of evidence according to the literature available at the time of the unlisted medication request and the line(s) of treatment previously received. The dependent variables calculated for each patient were progression-free survival (PFS) and overall survival (OS). PFS was calculated from the date of initiation of the unlisted medication to documented progression in the chart or death of any cause. This method differs from the Response Evaluation Criteria In Solid Tumors (RESIST 1.1) criteria used in clinical trials based on imaging. OS was calculated from the date of initiation of the unlisted medication to death of any cause. Additionally, patients who were lost to follow-up were censored.

Patients were excluded if they received the unlisted medication in the curative setting (adjuvant or neoadjuvant), never received an unlisted medication, were given the unlisted medication for different reasons other than cancer (e.g., if it was given for toxicity or complication reasons), or the medication was received outside of the time of the analysis window.

Appropriate ethics approval was given for this quality improvement project.

### 2.3. Outcome Description and Statistical Analysis

Outcomes in this trial are mainly descriptive, as formal statistical testing is impossible or not warranted. Statistical analysis was performed separately for each treatment-related group. A Kaplan–Meier survival analysis was performed to evaluate PFS and OS for patients. Statistical analysis between the curves was conducted when pertinent using the log-rank test in Prism software. The significance level was set at *p* < 0.05 in a two-sided test.

## 3. Results

### 3.1. Description of the Patients Receiving an Unlisted Anticancer Prescription

**Figure 1:**
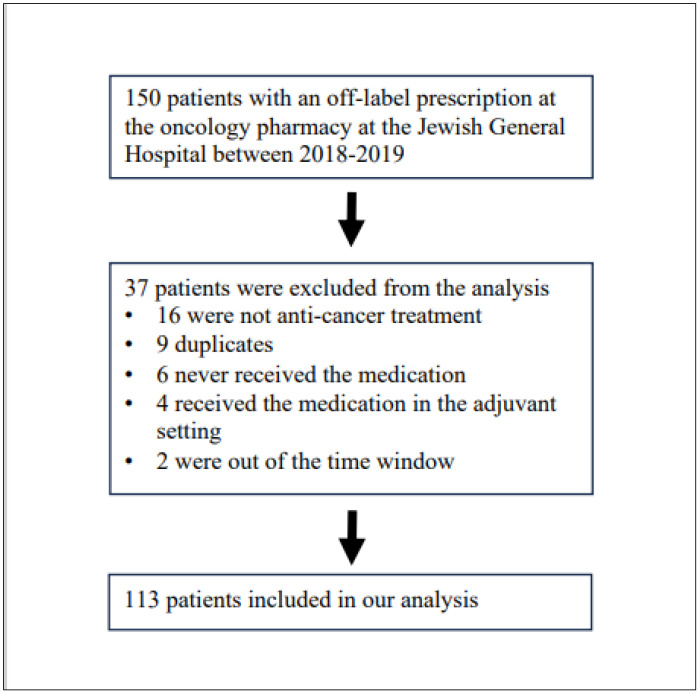
Consort Diagram.

From the 150 unlisted prescriptions at the oncology pharmacy, 113 patients received at least one dose of an unlisted anticancer prescription for uncurable cancer at the Jewish General Hospital between 2018 and 2019. A consort diagram is presented in Figure 1. The clinical characteristics of the study population are presented in Table 1, and the main features of treatments are presented in Table 2. The unlisted drugs prescribed are given in Table S1. The patients were evenly distributed between males and females, with most being between 51 and 70 years old. Moreover, indolent B-cell lymphoma was the main malignancy type in hematological cancers (25/54). The median follow-up was 60 months, counted from the date when the unlisted medication was administered.

### 3.2. Clinical Outcomes in Patients Receiving an Unlisted Anticancer Prescription

**Figure 2:**
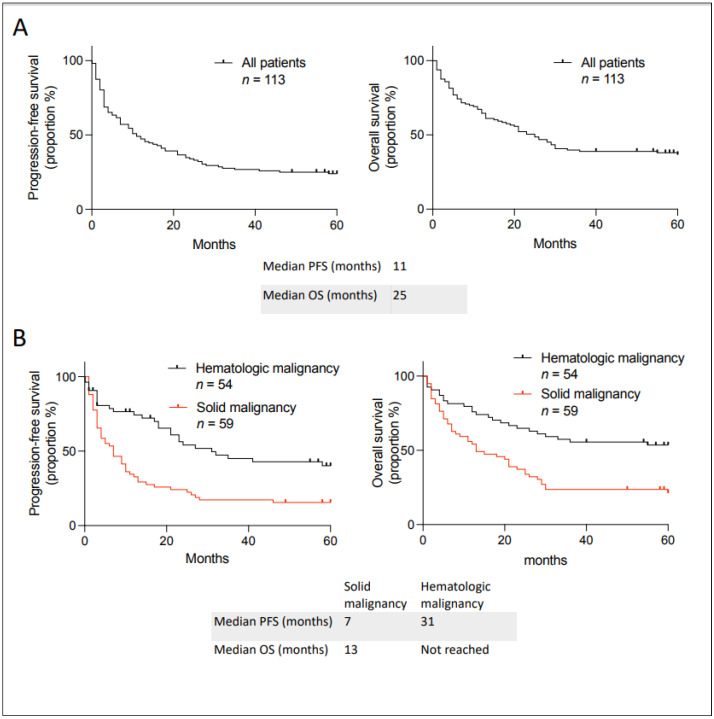
Kaplan–Meier curves describing PFS and OS in patients with incurable cancer receiving an unlisted anticancer treatment at the Jewish General Hospital between 2018–2019. (**A**) In all patients. (**B**) Classified between hematologic and solid malignancy. Median PFS and OS are given below the curves.

We first calculated the PFS and OS for all patients separated between solid and hematological malignancies (Figure 2). The median PFS and OS for all patients were 11 and 25 months, respectively (Figure 2A). Twenty-seven out of one hundred and thirteen patients did not have PFS events, and only four were still taking the unlisted medication. Indeed, immune checkpoint inhibitors were stopped after a complete response, and many treatments for hematologic malignancies only included a set number of cycles or treatment breaks, as patients might have stopped their medication. The four patients still taking the unlisted medication include two chronic myeloid leukemia patients taking asciminib, one case of ovarian cancer receiving bevacizumab, and one patient with Castleman disease taking siltuximab. Only two patients were lost to follow-up: an 87-year-old male with head and neck cutaneous squamous cell carcinoma with complete response to nivolumab after 50 months and a 64-year-old male with indolent B-cell lymphoma who progressed after receiving the unlisted maintenance rituximab after cytarabine-rituximab and was on acalabrutinib without evidence of progression after 40 months. This demonstrates that our institution rigorously follows incurable cancer patients when an unlisted medication is prescribed in order to facilitate such analyses. Moreover, three patients passed away evidently from an unrelated cause without evidence of progression of their cancer and were classified as having an event per the definition of PFS. Solid malignancy patients had numerically worse outcomes than hematological malignancy, likely reflecting a different natural history (Figure 2B; no statistical analysis was performed).

### 3.3. Analysis of Outcomes According to the Level of Evidence Supporting the Request

**Figure 3:**
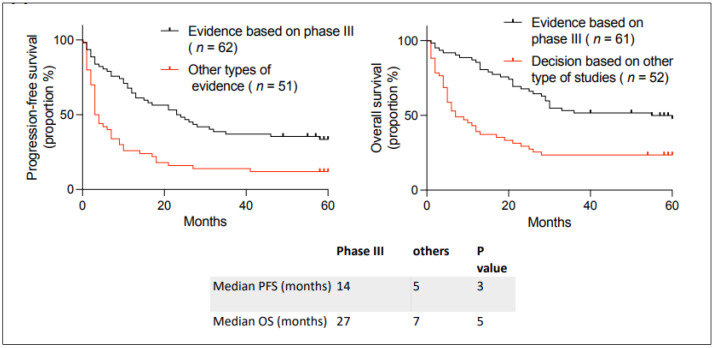
Kaplan–Meier curves describing PFS and OS in patients with incurable cancer receiving an unlisted anticancer treatment at the Jewish General Hospital between 2018–2019, depending on whether the request is based on a phase III clinical trial or other types of evidence. Median PFS and OS are given below the curves. The *p*-value between the curves was calculated using a two-sided log-rank test.

We then analyzed the data presented in the request “formulaire de demande d’utilisation d’un médicament pour des motifs de nécessité médicale particulière” according to the level of evidence for unlisted anticancer medications. Some requests were based on randomized phase III clinical trials, while others were based on other types of evidence. In other types of evidence, we included case series and phase I and II clinical trials. We analyzed the outcomes based on the level of evidence for the unlisted request (Figure 3), and we observed better outcomes if the unlisted medication request was based on randomized phase III clinical trials than those based on other types of evidence. This was true for hematologic and solid tumors (Supplementary Figure S1). The type of evidence and the reference for each request is given in Supplementary Table S2.

### 3.4. Analysis of Outcomes if the Medication Was Subsequently Reimbursed in Quebec

We then analyzed which of the various drugs requested using this special access program were subsequently reimbursed in the public healthcare system in Quebec, based on the recommendation of INESSS. A large proportion, 44%, of anticancer medications for incurable cancer were subsequently reimbursed in Quebec (Table 3). This means that for a large proportion of incurable cancer patients in which systemic therapy must be given without delay, the oncologist prescribed an unlisted treatment that was subsequently formally approved for reimbursement. This was particularly true for treatments supported by phase III clinical trials, in which 65% of medications were subsequently reimbursed. This suggests that high-quality data, particularly phase III clinical trials, commonly results in subsequent reimbursement.

### 3.5. Analysis of Outcomes According to the Type of Anticancer Medication

**Figure 4:**
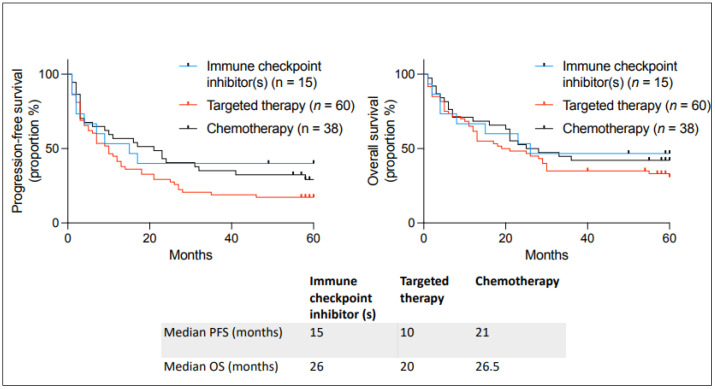
Kaplan–Meier curves describing PFS and OS in patients with incurable cancer receiving an unlisted anticancer treatment at the Jewish General Hospital between 2018–2019 and the type of unlisted medication used. Median PFS and OS are given below the curves.

Outcomes were also analyzed depending on the type of therapy used (Figure 4). We found no statistical difference between targeted therapy, immunotherapy, and chemotherapy. It is interesting to observe that many incurable patients on immune checkpoint inhibitors benefited from unlisted drug access; seven out of fifteen are still alive, emphasizing that patients had long-term benefits.

### 3.6. Analysis of Outcomes in Comparison to the Published Literature

We also analyzed the outcomes of our patients to those published in the literature, although the small number of patients treated for each indication made this challenging. We could only numerically describe outcomes without formal statistical comparison. Only groups of five patients or more treated with a drug for an indication were analyzed. In those patients, our real-world cohort had strikingly similar outcomes to published results (Table 4). This was true even if our solid tumors were heavily pretreated, usually more than in the formal clinical trial. This indicates that utilizing unlisted drug access achieved similar outcomes in a real-world setting as the published results from the literature. However, the outcomes described in Table 4 are only descriptive, and statistical comparison is impossible.

## 4. Discussion

In Quebec, a doctor can access a medication that is Health Canada-approved but not yet approved for reimbursement by the Ministry of Health and Social Services via the “formulaire de demande d’utilisation d’un médicament pour des motifs de nécessité médicale particulière”. In this study, we refer to such drugs as “unlisted” because they are not on Quebec’s reimbursement list. We conducted a retrospective analysis of anticancer medication in incurable cancer patients accessed via this mechanism. We analyzed data from 2018–2019 to ensure sufficient follow-up. Many patients were pretreated in the metastatic setting with a median of two lines of previous therapy. We found that patients who accessed medication through this mechanism had a median PFS of 11 months and OS of 25 months (Figure 2A). This is clinically meaningful for our patients since they had few effective therapeutic options when they came to this process, and therefore their life expectancy was limited. Groups of patients accessing an unlisted medication had similar outcomes to those in the literature, although statistical comparison is impossible (Table 4). Patients had particularly improved outcomes when the evidence behind the request was a phase III clinical trial compared to other types of evidence (Figure 3). However, even in other types of evidence, we still found significant benefits (response and estimated prolonged survival). Phase III clinical trials are long, expensive, and difficult to conduct in patients with rare types of cancer. This access to unlisted anticancer medication is important for treating patients with rare cancers. Furthermore, a large portion (44%) of anticancer drugs were later reimbursed in Quebec, which ultimately means that this access gave patients with incurable cancer access to approved medications earlier than the official listing process. Given the limited life expectancy of cancer patients, this pathway was probably their only way to access the medication. We only found one other report analyzing unlisted anticancer medication in a public healthcare system [17]. It came from an academic center in Spain, and they found lower median PFS and OS (5 and 11 months, respectively). Our study is difficult to compare to theirs, given the different treatments accessed and the tumor types. However, our longer PFS and OS could be due to our study being more recent (2018–2019) than theirs (2005–2015), with newer anticancer medications being more effective.

Moreover, three patients in our dataset treated with unlisted medications were described in the published literature as distinct case reports, which added this knowledge to cancer therapeutics. This is of crucial importance in an academic center. [18,19,20]. These patients include a melanoma patient who responded multiple times to immune checkpoint inhibition, a large diffuse B-cell lymphoma patient responding to the immune checkpoint inhibitor pembrolizumab, and a patient with Muir–Torre syndrome who stopped having new primary cancer after the initiation of pembrolizumab [18,19,20]. These cases were originally present in our dataset and were not included in this study because they were previously published.

The delays involved in new oncology drug reimbursement in Canada described earlier, including the sequential bureaucratic steps in drug efficacy and toxicity evaluation, followed by cost-analysis for reimbursement, leave patients with advanced cancers who might benefit from them waiting, with a likely detrimental effect on their clinical outcomes [4,6]. This study demonstrated that access to unlisted medications is important for some cancer patients. There are novel frameworks for providing timely patient access to new anticancer drugs in public healthcare settings while further evaluation is conducted, including conditional funding agreements, which could reduce the necessity for unlisted oncology treatments [4]. Conditional listing agreements typically allow a new drug to be prescribed shortly after market authorization, while further evaluation by authorities, including pricing discounts and the generation of real-world data, can later support the request [4].

Our study has numerous limitations. It is a survey of patients treated with unlisted drugs, so there is no comparator arm, and formal analysis of the statistical superiority of the unlisted medications is impossible. Moreover, the types of medications, lines of treatment, and primary cancers were diverse, which made formal comparison with the literature impossible, given the small number of patients treated for each indication. We compared the outcomes of our patients to the literature when at least five patients were treated with a medication for an indication (Table 4). In those groups, outcomes from our real-world dataset were similar to the published data available, indicating that we could reproduce outcomes in the real-world setting. Moreover, given that our dataset mainly comprised aggressive cancers, we are convinced that the observed PFS and OS with unlisted treatments are clinically meaningful; the patients were not likely to survive without an effective treatment. Our patients were duly followed, reflecting standards of practice in the real-world setting. All medications in this study were accessed through the “formulaire de demande d’utilisation d’un médicament pour des motifs de nécessité médicale particulière”. They were paid for by the hospital or by a pharmaceutical company special access program.

We also decided to include only patients with incurable cancer type in this analysis. However, it is known that unlisted medications can also significantly improve outcomes in the curative setting [21]. In another study using the same system to access pertuzumab in the neoadjuvant setting, which at the time was not yet approved in Quebec, in combination with neoadjuvant chemotherapy and trastuzumab in early human epidermal growth factor receptor 2 (HER2-positive) breast cancer, we demonstrated increased pathological complete response rate [21]. The study further showed that evaluating the cost of the entire trajectory of illness, instead of simply the drug cost, demonstrated that using this highly clinically effective though expensive drug was cost-neutral [21]. Eventually, after a delay of over a year, this was adopted as a new standard of practice and reimbursed in Quebec.

Some healthcare planners in Quebec have questioned and challenged the Medical Necessity Measure Program, potentially delaying or restricting the availability of certain anticancer medications for patients in need [22,23]. This could have significant implications for patients with incurable cancers who rely on unlisted treatments when no other options are available. By limiting access, patients might face prolonged periods without potentially beneficial therapies, leading to a deterioration in their health outcomes and quality of life. Furthermore, this limitation could hinder the adoption of innovative treatments that have shown promising results in real-world settings despite being unlisted. The restriction might discourage healthcare providers from prescribing unlisted drugs that could benefit patients, thereby limiting personalized care approaches. New thinking about the ‘value’ of novel therapies must be introduced into the current evaluation process [24].

The data presented here underscore the unlisted treatments’ potential efficacy, particularly because many unlisted medications were subsequently reimbursed. This emphasizes the importance, in any jurisdiction, of timely drug access for cancer patients. If these data were expanded to include a larger patient population, it would offer more robust and generalizable evidence, enhancing the credibility and reliability of the findings. We are working with other institutions to expand the data and the experience at several hospitals. Such comprehensive real-world evidence could be a valuable resource for decision-making institutions like INESSS, enabling them to make more informed and expedited treatment approval decisions.

Additionally, the information captured in this paper will serve as a foundation for broader analyses regarding the approval processes for drugs, especially those used unlisted, and it highlights the importance of incorporating real-world data into the evaluation and approval of new treatments, thereby promoting a more comprehensive and pragmatic approach to drug regulation. These foundational data can stimulate further studies and analyses exploring unlisted drug use, efficacy, and safety, ultimately contributing to a more dynamic and responsive healthcare system. By advocating for integrating real-world evidence into approval processes, this paper paves the way for more timely and equitable access to innovative therapies for patients with urgent medical needs. As precision treatment becomes the standard of more effective cancer treatments, systems like this access program must continue and expand while maintaining rigorous standards and consistent reviews so clinical investigators and clinicians can access the right drug for the right patient at the right time.

## 5. Conclusions

The real-world outcomes of patients receiving an unlisted anticancer medication emphasize the importance of timely oncology drug approval. Even if the lack of a comparator arm did not enable us to conclude the superiority of the unlisted anticancer drugs, we observed clinically meaningful activity of unlisted anticancer medications in an academic center in the province of Quebec. Moreover, a large proportion of the medications accessed were subsequently reimbursed after some delay in the province of Quebec, giving access to medications to patients who could not wait. Timely oncology drug access could be achieved through conditional funding agreements. Furthermore, real-world data are of crucial importance in the drug approval process.

## Figures and Tables

**Table 1:** Characteristics of patients with an incurable/metastatic malignancy who received an uncovered anticancer therapy at the Jewish General Hospital between 2018–2019.

Features	N (%)
**Sex**	
Male	57 (50%)
Female	56 (50%)
**Age (years)**	
18–50	18 (16%)
51–71	62 (55%)
≥71	35 (31%)
**Type of cancer**	
**Solid malignancy**	59 (52%)
Breast	10 (9%)
Pancreatic	8 (7%)
Colorectal	7 (6%)
Ovarian	6 (5%)
Skin, melanoma	6 (5%)
Renal cell	6 (5%)
Lung cancer, non-small cell lung cancer	4 (4%)
Skin, squamous carcinoma	3 (3%)
Head and neck, squamous carcinoma	2 (2%)
Bladder	2 (2%)
Angiosarcoma, prostate, leiomyosarcoma, cervix, endometrium	1 (1%) each
**Hematological malignancy**	54 (48%)
Indolent B-cell lymphoma	25 (22%)
Multiple myeloma	10 (9%)
Acute myeloid leukemia	5 (4%)
Hodgkin lymphoma	3 (3%)
Chronic myelocytic leukemia	2 (2%)
Large B-cell lymphoma	2 (2%)
Myelodysplastic syndrome	2 (2%)
Blastic plasmacytoid dendritic cell neoplasm, lymphoplasmacytic lymphoma, peripheral T cell lymphoma, other monoclonal plasma cell disorder, Castleman disease	1 (1%) each

**Table 2:** Features of the 113 unlisted anticancer treatments given for incurable cancers at the Jewish General Hospital in 2018–2019.

Variable	N (%)
**Type of treatment**	
Targeted therapy	60 (53%)
Chemotherapy	38 (34%)
Immune checkpoint inhibitor	15 (13%)
**Evidence supporting the request**	
Phase III randomized clinical trial	62 (55%)
Others	51 (45%)
**Subsequently reimbursed by the public health system in Quebec**	
Yes	50 (44%)
No	63 (56%)
**Line of therapy**	
First line	31 (27%)
Second line	24 (21%)
Third line	26 (23%)
Fourth line	17 (15%)
Fifth line	6 (5%)
Sixth to tenth line	9 (8%)

**Table 3:** Analysis of unlisted oncology anticancer oncology prescriptions for incurable cancer at the Jewish General Hospital that were subsequently reimbursed in the province of Quebec and according to the evidence supporting the request.

Types of Evidence	All Types of Evidence *N* = 113 (100%)	Phase III Clinical Trial *N* = 62 (54.8%)	Other Types of Evidence *N* = 51 (45%)
Subsequently reimbursed	50 (44%)	40 (65%)	10 (20%)

**Table 4:** Outcomes in patients with incurable cancer receiving an unlisted anticancer treatment at the Jewish General Hospital between 2018–2019 and in the literature. Only groups containing more than five patients treated with a drug for an indication are shown for comparison.

Number of Patients in Our Real-World Cohort	Type of Cancer	Previous Line of Treatment	Drug Name	Type of Comparator Trial, Reference	Outcomes in the Comparator Trial	Outcomes in Our Unlisted Cohort
14	Indolent B-cell lymphoma	0	Bendamustine-rituximab	Phase III, [12]	PFS at 5 years: 65%	PFS at 5 years: 9/14 (64%)
6	Melanoma	0	Ipilimumab-nivolumab	Phase III, [13]	Median PFS: 11.5 months Median OS: not reached	Median PFS: 12 months Median OS: not reached (3/6 still alive at five years)
5	Breast cancer	4 to 9	Liposomal doxorubicin	Retrospective analysis, [14]	Median PFS: 5.8 months Median OS: 14.2 months	Median PFS: 3 months Median OS: 6 months
5	Renal cell cancer	2 to 3	Cabozantinib	Phase III, [15]	Median PFS: 7.4 months Median OS: 21.4 months	Median PFS: 7 months Median OS: 28 months
5	Pancreatic cancer	2 to 3	Liposomal irinotecan with 5-FU *	Retrospective data, [16]	Median PFS: 3.5 months Median OS: 9.4 months	Median PFS: 3 months Median OS: 6 months

* 5-FU = fluorouracil.

## Data Availability

The original data presented in the study are included in the article/Supplementary Materials, further inquiries can be directed to the corresponding author/s.

## Supplementary Materials

The following supporting information can be downloaded at: https://www.mdpi.com/article/10.3390/curroncol31100440/s1, Table S1: drugs name of the 113 uncovered medication; Figure S1: Kaplan-Meier curves describing PFS and OS in patients with incurable cancer receiving an uncovered anticancer treatment at the Jewish General Hospital between 2018–2019 depending if the request based on phase III clinical trial or other types of evidence divided between solid (**A**) and hematologic (**B**) malignancies. Median PFS and OS are given below the curves. The *p*-value between the curves was conducted using a two-sided log-rank test; Table S2: specific medication given, the number of previous lines of treatment, and the evidence supporting the uncovered request.
