# Supplementary material for: Real-World Outcomes of Incurable Cancer Patients Treated with Unlisted Anticancer Treatments in an Academic Center in Quebec, Canada
Source: Curr Oncol. 2024 Oct 1;31(10):5908–18. doi: 10.3390/curroncol31100440 (PMC11506447; doi:10.3390/curroncol31100440)
Supplement: Supplementary file 1 [file curroncol-31-00440-s001.zip › curroncol-3190757-supplementary.pdf]

Real-world outcomes of incurable cancer patients treated with an uncovered anticancer treatment in an Academic Centre in Quebec, Canada

Supplementary data

Table S1 : drugs name of the 113 uncovered medication

**Variable**	**N**
Name of the drugs solid malignancy	
Liposomal irinotecan	8
Liposomal doxorubicin	7
Ipilimumab (combined with nivolumab)	7
Bevacizumab	7
Panitumumab	6
Cabozantinib	5
Pertuzumab	4
Pembrolizumab	3
Lorlatinib	2
Cemiplimab	2
Nivolumab	2
Alectinib	1
Cabazitaxel	1
Cetuximab	1
Crizotinib	1
Olaratumab	1
Thiotepa	1
Name of the drugs hematological malignancy	
Bendamustine-rituximab	18
Carfilzomib	5
Bortezomib	4
Azacitidine-Venetoclax	4
Rituximab	4
Obinutuzimab-bendamustine	3

Real-world outcomes of incurable cancer patients treated with an uncovered anticancer treatment in an Academic Centre in Quebec, Canada

Supplementary data

Brentuximab	2
Daratumumab	2
Azacitidine	2
Asciminib	2
Alectinib	1
Arsenic trioxide	1
Entospletinib	1
Inotuzumab ozogamicin	1
Siltuximab	1
Pembrolizumab	1
Romidepsin	1
Venetoclax	1

Real-world outcomes of incurable cancer patients treated with an uncovered anticancer treatment in an Academic Centre in Quebec, Canada

Supplementary data

A

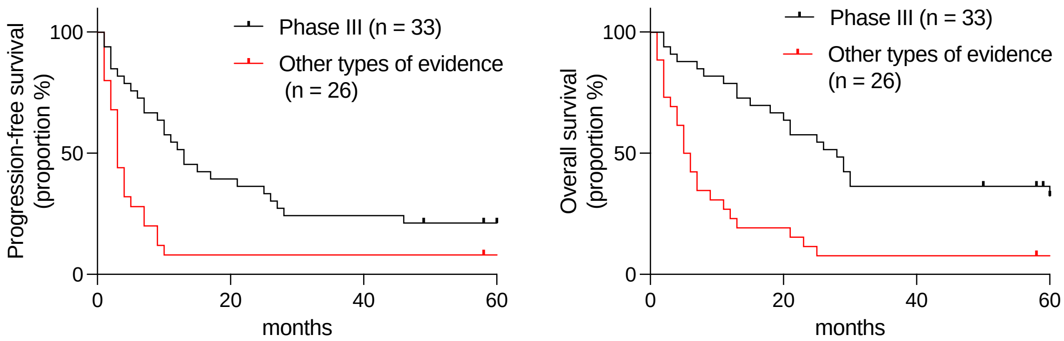

	Phase III	Other	P- value
Median PFS (months)	13	3	0.0008
Median OS (months)	28	5.5	<0.0001

B

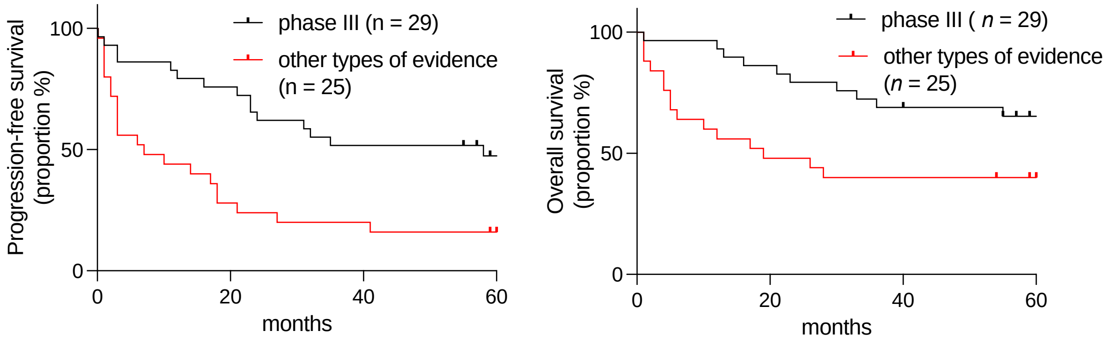

	Phase III	Other	P- value
Median PFS (months)	58	7	0.0014
Median OS (months)	Not reached	19	0.0184

Figure S1: Kaplan-Meier curves describing PFS and OS in patients with incurable cancer receiving an uncovered anticancer treatment at the Jewish General Hospital between 2018-2019 depending if the request based on phase III clinical trial or other types of evidence divided between solid (A) and hematologic (B) malignancies. Median PFS and OS are given below the curves. The P-value between the curves was conducted using a two-sided log-rank test.

Table S2: specific medication given, the number of previous lines of treatment, and the evidence supporting the uncovered request

Real-world outcomes of incurable cancer patients treated with an uncovered anticancer treatment in an Academic Centre in Quebec, Canada

Supplementary data

Number of patient(s)	Type of cancer	Previous line of treatment	Drug name	Ref	Type of trial
1	NSCLC	0	alectinib	[1]	phase III
1	Alk+ large B-cell lymphoma	1	alectinib	[2]	phase II
1	Acute promyelocytic Leukemiia	0	Arsenic trioxide-ATRA	[3]	phase III
2	chronic myelocytic leukemia	2 to 4	Asciminib	[4]	Phase I/II
2	Transplant eligible myelodysplasic syndrome	0	Azacitadine	[5]	phase III
3	Acute myeloid leukemia relapse	1 to 5	Venetoclax-azacitidine	[6]	Retrospective data
1	Hodgkin lymphoma	2	brentuximab-vendotin	[7]	phase III
4	ovarian cancer	2 to 3	bevacizumab	[8]	phase III
1	Blastic plasmacytoid dendritic cell neoplasm	2	Azacitidine-Venetoclax		tumor board recommandation
1	other monoclonal plasma cell disorder	1	Bortezomib	[9]	Retrospective data
1	Acute myeloid leukemia relapse	2	Entospletinib	[10]	Phase I/II
1	B Cell Lymphoma	3	pembrolizumab	[11]	Retrospective data
14	Indolent B-cell lymphoma	0	bendamustine-rituximab	[12]	phase III
1	ovarian cancer	unknown, platinum	carboplatin-liposomal doxorubicine	[13]	phase III

Real-world outcomes of incurable cancer patients treated with an uncovered anticancer treatment in an Academic Centre in Quebec, Canada

Supplementary data

		sensitive			
1	angiosarcoma	0	liposomal doxorubicin	[14]	Phase II
6	melanoma	0	ipilimumab-nivolumab	[15]	phase III
2	Head and Neck squamous	0	Nivolumab	[16]	phase III
1	Renal cell carcinoma	0	Ipilimumab-nivolumab	[17]	phase III
1	prostate	3	cabazitazel carboplatin	[18]	phase I/II
3	mantle cell lymphoma	1	bendamustine-rituximab	[19]	Phase I/II
1	mantle cell lymphoma	3	venetoclax	[20]	Phase I/II
2	NSCLC	2	lorlatinib	[21]	phase II
3	multiple myeloma	3 to 5	carfilzomib	[22]	phase III
5	breast cancer	4 to 9	liposomal doxorubicin	[23]	Retrospective analysis
3	B cell indolent lymphoma	1 to 2	Obinutuzimab-Bendamustine	[24]	phase III
3	multiple myeloma	3 to 5	Bortezomib	[25]	retrospective data
1	leukemia	1	Inotuzumab Ozogamicin	[26]	expert consensus
1	colorectal cancer with deficient mismatch repair	1	pembrolizumab	[27]	phase II
1	bladder cancer	1	pembrolizumab	[28]	phase III
1	cervical cancer metastatic	2	bevacizumab	[29]	phase III
2	mantle cell lymphoma	1 to 2	rituximab	[30]	phase III
5	Renal cell carcinoma	2 to 3	cabozantinib	[31]	phase III
2	skin, squamous	0 to 1	cemiplimab	[32]	Phase I/II
1	head and Neck squamous	2	cetuximab	[33]	Phase II
1	hodgkin lymphoma	4	bendamustine	[34]	Phase I/II

5	pancreatic	2	liposomal irinotecan with 5-Fu	[35]	retrospective data
3	pancreatic	1	liposomal irinotecan with 5-Fu	[36]	phase III
1	breast cancer	1	pertuzumab	[37]	phase III
3	breast cancer	2 to 8	pertuzumab	[38]	retrospective data
1	peripheral cutaneous T cell lymphoma	2	Romidepsin	[39]	Phase I/II
1	multiple myeloma	3	Daratumumab, cyclophospham ide, dexametasone	[40]	Phase II
1	Indolent B-cell lymphoma	1	rituximab	[41]	phase III
1	ovarian cancer	2	bevacizumab olaparib	[42]	phase III
3	colorectal cancer	0 to 1	panitumumab	[43]	phase II
1	multiple myeloma	6	Daratumumab, bortezomib, dexametasone	Tumor board recommandat ion (Bortezomib refractory)	
1	leiomyosarcom a	1	olatumab-doxorubicine	[44]	phase II
1	colorectal cancer	1	panitumumab-avastin	[45]	phase III
1	lymphoplasmoc ytic lymphoma	0	rituximab-cvp	[46]	retrospective data
1	Castleman disease	1	siltuximab	[47]	phase III
1	hodgkin lymphoma	3	brentuximab-vendotin	[48]	phase II
2	multiple myeloma	3 to 6	carfilzomib with thalidomide and dexametasone	tumor board consensus	

Real-world outcomes of incurable cancer patients treated with an uncovered anticancer treatment in an Academic Centre in Quebec, Canada

Supplementary data

1	ovarian cancer	5	bevacizumab (with topotecan)	[49]	phase II
1	NSCLC	0	crizotinib	[50]	phase I
1	colorectal cancer	2	panitumumab (with capecitabine)	[51]	phase II
1	colorectal cancer	2	panitumumab vemurafenib	[52]	pilot trial
1	breast cancer	3	thiotepa intrathecal	[53]	retrospective data
1	bladder cancer	unknow n	pembrolizumab	[54]	phase II

1. Peters, S.; Camidge, D.R.; Shaw, A.T.; Gadgeel, S.; Ahn, J.S.; Kim, D.-W.; Ou, S.-H.I.; Pérol, M.; Dziadziuszko, R.; Rosell, R. Alectinib versus crizotinib in untreated ALK-positive non–small-cell lung cancer. *New England Journal of Medicine* **2017**, *377*, 829-838.
2. Reed, D.R.; Hall, R.D.; Gentzler, R.D.; Volodin, L.; Douvas, M.G.; Portell, C.A. Treatment of refractory ALK rearranged anaplastic large cell lymphoma with alectinib. *Clinical Lymphoma Myeloma and Leukemia* **2019**, *19*, e247-e250.
3. Burnett, A.K.; Russell, N.H.; Hills, R.K.; Bowen, D.; Kell, J.; Knapper, S.; Morgan, Y.G.; Lok, J.; Grech, A.; Jones, G. Arsenic trioxide and all-trans retinoic acid treatment for acute promyelocytic leukaemia in all risk groups (AML17): results of a randomised, controlled, phase 3 trial. *The lancet oncology* **2015**, *16*, 1295-1305.
4. Hughes, T.P.; Mauro, M.J.; Cortes, J.E.; Minami, H.; Rea, D.; DeAngelo, D.J.; Breccia, M.; Goh, Y.-T.; Talpaz, M.; Hochhaus, A. Asciminib in chronic myeloid leukemia after ABL kinase inhibitor failure. *New England Journal of Medicine* **2019**, *381*, 2315-2326.
5. Voso, M.; Leone, G.; Piciocchi, A.; Fianchi, L.; Santarone, S.; Candoni, A.; Criscuolo, M.; Masciulli, A.; Cerqui, E.; Molteni, A. Feasibility of allogeneic stem-cell transplantation after azacitidine bridge in higher-risk myelodysplastic syndromes and low blast count acute myeloid leukemia: results of the BMT-AZA prospective study. *Annals of Oncology* **2017**, *28*, 1547-1553.
6. Tenold, M.; Moskoff, B.; Benjamin, D.; Jonas, B.A. Retrospective analysis of adults with acute myeloid leukemia treated with venetoclax plus hypomethylating agents at a comprehensive cancer center. *Blood* **2018**, *132*, 1424.
7. Moskowitz, C.H.; Walewski, J.; Nademanee, A.; Masszi, T.; Agura, E.; Holowiecki, J.; Abidi, M.H.; Chen, A.I.; Stiff, P.; Viviani, S. Five-year PFS from the AETHERA trial

of brentuximab vedotin for Hodgkin lymphoma at high risk of progression or relapse. *Blood, The Journal of the American Society of Hematology* **2018**, *132*, 2639-2642.
8. Bamias, A.; Gibbs, E.; Lee, C.K.; Davies, L.; Dimopoulos, M.; Zagouri, F.; Veillard, A.-S.; Kosse, J.; Santaballa, A.; Mirza, M. Bevacizumab with or after chemotherapy for platinum-resistant recurrent ovarian cancer: exploratory analyses of the AURELIA trial. *Annals of Oncology* **2017**, *28*, 1842-1848.
9. Kourelis, T.; Kapoor, P.; Dingli, D.; Stewart, A.K.; Buadi, F.; Gertz, M.A.; Lacy, M.; Kumar, S.; Gonsalves, W.I.; Go, R.S. The use of proteasome inhibitors among patients with POEMS syndrome. **2017**.
10. Walker, A.R.; Bhatnagar, B.; Marcondes, A.M.Q.; DiPaolo, J.; Vasu, S.; Mims, A.S.; Klisovic, R.B.; Walsh, K.J.; Canning, R.; Behbehani, G.K. Interim results of a phase 1b/2 study of entospletinib (GS-9973) monotherapy and in combination with chemotherapy in patients with acute myeloid leukemia. **2016**.
11. Kim, S.-J.; Hyeon, J.; Cho, I.; Ko, Y.H.; Kim, W.S. Comparison of efficacy of pembrolizumab between epstein-barr virus–positive and–negative relapsed or refractory non-hodgkin lymphomas. *Cancer Res Treat* **2019**, *51*, 611-622.
12. Flinn, I.W.; Van Der Jagt, R.; Kahl, B.; Wood, P.; Hawkins, T.; MacDonald, D.; Simpson, D.; Kolibaba, K.; Issa, S.; Chang, J. First-line treatment of patients with indolent non-Hodgkin lymphoma or mantle-cell lymphoma with bendamustine plus rituximab versus R-CHOP or R-CVP: results of the BRIGHT 5-year follow-up study. *Journal of Clinical Oncology* **2019**, *37*, 984.
13. Wagner, U.; Marth, C.; Largillier, R.; Kaern, J.; Brown, C.; Heywood, M.; Bonaventura, T.; Vergote, I.; Piccirillo, M.; Fossati, R. Final overall survival results of phase III GCIG CALYPSO trial of pegylated liposomal doxorubicin and carboplatin vs paclitaxel and carboplatin in platinum-sensitive ovarian cancer patients. *British journal of cancer* **2012**, *107*, 588-591.
14. Judson, I.; Radford, J.A.; Harris, M.; Blay, J.-Y.; van Hoesel, Q.; Le Cesne, A.; Van Oosterom, A.; Clemons, M.; Kamby, C.; Hermans, C. Randomised phase II trial of pegylated liposomal doxorubicin (DOXIL®/CAELYX®) versus doxorubicin in the treatment of advanced or metastatic soft tissue sarcoma: a study by the EORTC Soft Tissue and Bone Sarcoma Group. *European Journal of Cancer* **2001**, *37*, 870-877.
15. Hodi, F.S.; Chiarion-Sileni, V.; Gonzalez, R.; Grob, J.-J.; Rutkowski, P.; Cowey, C.L.; Lao, C.D.; Schadendorf, D.; Wagstaff, J.; Dummer, R. Nivolumab plus ipilimumab or nivolumab alone versus ipilimumab alone in advanced melanoma (CheckMate 067): 4-year outcomes of a multicentre, randomised, phase 3 trial. *The Lancet Oncology* **2018**, *19*, 1480-1492.
16. Ferris, R.L.; Blumenschein Jr, G.; Fayette, J.; Guigay, J.; Colevas, A.D.; Licitra, L.; Harrington, K.; Kasper, S.; Vokes, E.E.; Even, C. Nivolumab for recurrent

squamous-cell carcinoma of the head and neck. *New England Journal of Medicine* **2016**, *375*, 1856-1867.
17. Escudier, B.; Tannir, N.; McDermott, D.; Frontera, O.; Melichar, B.; Plimack, E.; Barthelemy, P.; George, S.; Neiman, V.; Porta, C. CheckMate 214: efficacy and safety of nivolumab+ ipilimumab (N+ I) v sunitinib (S) for treatment-naïve advanced or metastatic renal cell carcinoma (mRCC), including IMDC risk and PD-L1 expression subgroups. *Annals of Oncology* **2017**, *28*, v621-v622.
18. Corn, P.G.; Heath, E.I.; Zurita, A.; Ramesh, N.; Xiao, L.; Sei, E.; Li-Ning-Tapia, E.; Tu, S.-M.; Subudhi, S.K.; Wang, J. Cabazitaxel plus carboplatin for the treatment of men with metastatic castration-resistant prostate cancers: a randomised, open-label, phase 1–2 trial. *The lancet oncology* **2019**, *20*, 1432-1443.
19. Czuczman, M.S.; Goy, A.; Lamonica, D.; Graf, D.; Munteanu, M.; Van der Jagt, R. Phase II study of bendamustine combined with rituximab in relapsed/refractory mantle cell lymphoma: efficacy, tolerability, and safety findings. *Annals of hematology* **2015**, *94*, 2025-2032.
20. Davids, M.S.; Roberts, A.W.; Seymour, J.F.; Pagel, J.M.; Kahl, B.S.; Wierda, W.G.; Puvvada, S.; Kipps, T.J.; Anderson, M.A.; Salem, A.H. Phase I first-in-human study of venetoclax in patients with relapsed or refractory non-Hodgkin lymphoma. *Journal of Clinical Oncology* **2017**, *35*, 826.
21. Solomon, B.J.; Besse, B.; Bauer, T.M.; Felip, E.; Soo, R.A.; Camidge, D.R.; Chiari, R.; Bearz, A.; Lin, C.-C.; Gadgeel, S.M. Lorlatinib in patients with ALK-positive non-small-cell lung cancer: results from a global phase 2 study. *The Lancet Oncology* **2018**, *19*, 1654-1667.
22. Dimopoulos, M.A.; Goldschmidt, H.; Niesvizky, R.; Joshua, D.; Chng, W.-J.; Oriol, A.; Orlowski, R.Z.; Ludwig, H.; Facon, T.; Hajek, R. Carfilzomib or bortezomib in relapsed or refractory multiple myeloma (ENDEAVOR): an interim overall survival analysis of an open-label, randomised, phase 3 trial. *The Lancet Oncology* **2017**, *18*, 1327-1337.
23. Fiegl, M.; Mlineritsch, B.; Hubalek, M.; Bartsch, R.; Pluschnig, U.; Steger, G.G. Single-agent pegylated liposomal doxorubicin (PLD) in the treatment of metastatic breast cancer: results of an Austrian observational trial. *BMC cancer* **2011**, *11*, 1-9.
24. Sehn, L.H.; Chua, N.; Mayer, J.; Dueck, G.; Trněný, M.; Bouabdallah, K.; Fowler, N.; Delwail, V.; Press, O.; Salles, G. Obinutuzumab plus bendamustine versus bendamustine monotherapy in patients with rituximab-refractory indolent non-Hodgkin lymphoma (GADOLIN): a randomised, controlled, open-label, multicentre, phase 3 trial. *The Lancet Oncology* **2016**, *17*, 1081-1093.
25. Min, C.-K.; Lee, M.-J.; Eom, K.-S.; Lee, S.; Lee, J.-W.; Min, W.-S.; Kim, C.-C.; Kim, M.; Lim, J.; Kim, Y. Bortezomib in combination with conventional chemotherapeutic agents for multiple myeloma compared with bortezomib alone. *Japanese journal of clinical oncology* **2007**, *37*, 961-968.

26. Kantarjian, H.M.; DeAngelo, D.J.; Stelljes, M.; Martinelli, G.; Liedtke, M.; Stock, W.; Gökbuget, N.; O'Brien, S.; Wang, K.; Wang, T. Inotuzumab ozogamicin versus standard therapy for acute lymphoblastic leukemia. *New England Journal of Medicine* **2016**, *375*, 740-753.
27. Diaz, L.; Marabelle, A.; Kim, T.; Geva, R.; Van Cutsem, E.; André, T.; Ascierto, P.; Maio, M.; Delord, J.; Gottfried, M. Efficacy of pembrolizumab in phase 2 KEYNOTE-164 and KEYNOTE-158 studies of microsatellite instability high cancers. *Annals of Oncology* **2017**, *28*, v128-v129.
28. Bajorin, D.F.; De Wit, R.; Vaughn, D.J.; Fradet, Y.; Lee, J.-L.; Fong, L.; Vogelzang, N.J.; Climent, M.Á.; Petrylak, D.P.; Choueiri, T.K. Planned survival analysis from KEYNOTE-045: Phase 3, open-label study of pembrolizumab (pembro) versus paclitaxel, docetaxel, or vinflunine in recurrent, advanced urothelial cancer (UC). **2017**.
29. Tewari, K.S.; Sill, M.W.; Penson, R.T.; Huang, H.; Ramondetta, L.M.; Landrum, L.M.; Oaknin, A.; Reid, T.J.; Leitao, M.M.; Michael, H.E. Bevacizumab for advanced cervical cancer: final overall survival and adverse event analysis of a randomised, controlled, open-label, phase 3 trial (Gynecologic Oncology Group 240). *The Lancet* **2017**, *390*, 1654-1663.
30. Le Gouill, S.; Thieblemont, C.; Oberic, L.; Moreau, A.; Bouabdallah, K.; Gyan, E.; Damaj, G.; Ribrag, V.; Feugier, P.; Casasnovas, O. Rituximab maintenance after autologous stem cell transplantation prolongs survival in younger patients with mantle cell lymphoma: final results of the randomized phase 3 LyMa trial of the Lysa/Goelams Group. *Blood* **2016**, *128*, 145.
31. Choueiri, T.K.; Escudier, B.; Powles, T.; Tannir, N.M.; Mainwaring, P.N.; Rini, B.I.; Hammers, H.J.; Donskov, F.; Roth, B.J.; Peltola, K. Cabozantinib versus everolimus in advanced renal cell carcinoma (METEOR): final results from a randomised, open-label, phase 3 trial. *The lancet oncology* **2016**, *17*, 917-927.
32. Migden, M.R.; Rischin, D.; Schmults, C.D.; Guminski, A.; Hauschild, A.; Lewis, K.D.; Chung, C.H.; Hernandez-Aya, L.; Lim, A.M.; Chang, A.L.S. PD-1 blockade with cemiplimab in advanced cutaneous squamous-cell carcinoma. *New England Journal of Medicine* **2018**, *379*, 341-351.
33. Vermorken, J.B.; Trigo, J.; Hitt, R.; Koralewski, P.; Diaz-Rubio, E.; Rolland, F.; Knecht, R.; Amellal, N.; Schueler, A.; Baselga, J. Open-label, uncontrolled, multicenter phase II study to evaluate the efficacy and toxicity of cetuximab as a single agent in patients with recurrent and/or metastatic squamous cell carcinoma of the. *Journal of clinical oncology* **2007**, *25*, 2171-2177.
34. Moskowitz, A.J.; Hamlin Jr, P.A.; Perales, M.-A.; Gerecitano, J.; Horwitz, S.M.; Matasar, M.J.; Noy, A.; Palomba, M.L.; Portlock, C.S.; Straus, D.J. Phase II study of bendamustine in relapsed and refractory Hodgkin lymphoma. *Journal of Clinical Oncology* **2013**, *31*, 456.

35. Yoo, C.; Im, H.-S.; Kim, K.-p.; Oh, D.-Y.; Lee, K.-H.; Chon, H.J.; Kim, J.H.; Kang, M.; Kim, I.; Lee, G.J. Real-world efficacy and safety of liposomal irinotecan plus fluorouracil/leucovorin in patients with metastatic pancreatic adenocarcinoma: a study by the Korean Cancer Study Group. *Therapeutic advances in medical oncology* **2019**, *11*, 1758835919871126.
36. Wang-Gillam, A.; Li, C.-P.; Bodoky, G.; Dean, A.; Shan, Y.-S.; Jameson, G.; Macarulla, T.; Lee, K.-H.; Cunningham, D.; Blanc, J.F. Nanoliposomal irinotecan with fluorouracil and folinic acid in metastatic pancreatic cancer after previous gemcitabine-based therapy (NAPOLI-1): a global, randomised, open-label, phase 3 trial. *The Lancet* **2016**, *387*, 545-557.
37. Urruticoechea, A.; Rizwanullah, M.; Im, S.-A.; Sanchez-Ruiz, A.C.; Lang, I.; Tomasello, G.; Douthwaite, H.; Badovinac Crnjevic, T.; Heeson, S.; Eng-Wong, J. PHEREXA: A phase III study of trastuzumab (H)+ capecitabine (X)±pertuzumab (P) for patients (pts) who progressed during/after one line of H-based therapy in the HER2-positive metastatic breast cancer (MBC) setting. **2016**.
38. Bergin, A.R.; Luen, S.J.; Savas, P.; Boolell, V.; Cho, D.; Lynch, J.; Nott, L.; Stuart-Harris, R.; Teo, L.N.; Yap, S.Y. Efficacy of late line pertuzumab with trastuzumab and chemotherapy in HER2-positive metastatic breast cancer: An Australian case series. *Asia-Pacific Journal of Clinical Oncology* **2019**, *15*, 377-382.
39. Piekarz, R.L.; Frye, R.; Prince, H.M.; Kirschbaum, M.H.; Zain, J.; Allen, S.L.; Jaffe, E.S.; Ling, A.; Turner, M.; Peer, C.J. Phase 2 trial of romidepsin in patients with peripheral T-cell lymphoma. *Blood, The Journal of the American Society of Hematology* **2011**, *117*, 5827-5834.
40. Sebag, M.; Bahlis, N.; Venner, C.P.; McCurdy, A.; Kouroukis, C.T.; Shustik, J.; White, D.J.; Kotb, R.; Stakiw, J.; Laferriere, N.B. A randomized phase II, open label, study of daratumumab, weekly low-dose oral dexamethasone and cyclophosphamide with or without pomalidomide in patients with relapsed and refractory multiple myeloma. *Blood* **2019**, *134*, 3121.
41. Salles, G.; Seymour, J.F.; Offner, F.; López-Guillermo, A.; Belada, D.; Xerri, L.; Feugier, P.; Bouabdallah, R.; Catalano, J.V.; Brice, P. Rituximab maintenance for 2 years in patients with high tumour burden follicular lymphoma responding to rituximab plus chemotherapy (PRIMA): a phase 3, randomised controlled trial. *The Lancet* **2011**, *377*, 42-51.
42. Ray-Coquard, I.; Selle, F.; Harter, P.; Cropet, C.; Marth, C.; Vergote, I.; Fujiwara, K.; Gonzalez-Martin, A.; Pignata, S.; Colombo, N. PAOLA-1: An ENGOT/GCIG phase III trial of olaparib versus placebo combined with bevacizumab as maintenance treatment in patients with advanced ovarian cancer following first-line platinum-based chemotherapy plus bevacizumab. **2016**.
43. Sastre, J.; Massuti, B.; Pulido, G.; Guillén-Ponce, C.; Benavides, M.; Manzano, J.; Reboredo, M.; Rivera, F.; Grávalos, C.; Safont, M. First-line single-agent panitumumab in frail elderly patients with wild-type KRAS metastatic colorectal

cancer and poor prognostic factors: a phase II study of the Spanish Cooperative Group for the Treatment of Digestive Tumours. *European Journal of Cancer* **2015**, *51*, 1371-1380.

44. Tap, W.D.; Jones, R.L.; Van Tine, B.A.; Chmielowski, B.; Elias, A.D.; Adkins, D.; Agulnik, M.; Cooney, M.M.; Livingston, M.B.; Pennock, G. Olaratumab and doxorubicin versus doxorubicin alone for treatment of soft-tissue sarcoma: an open-label phase 1b and randomised phase 2 trial. *The Lancet* **2016**, *388*, 488-497.
45. Douillard, J.-Y.; Oliner, K.S.; Siena, S.; Tabernero, J.; Burkes, R.; Barugel, M.; Humblet, Y.; Bodoky, G.; Cunningham, D.; Jassem, J. Panitumumab–FOLFOX4 treatment and RAS mutations in colorectal cancer. *New England Journal of Medicine* **2013**, *369*, 1023-1034.
46. Ioakimidis, L.; Patterson, C.J.; Hunter, Z.R.; Soumerai, J.D.; Manning, R.J.; Turnbull, B.; Sheehy, P.; Treon, S.P. Comparative outcomes following CP-R, CVP-R, and CHOP-R in Waldenström's macroglobulinemia. *Clinical Lymphoma and Myeloma* **2009**, *9*, 62-66.
47. Van Rhee, F.; Wong, R.S.; Munshi, N.; Rossi, J.-F.; Ke, X.-Y.; Fosså, A.; Simpson, D.; Capra, M.; Liu, T.; Hsieh, R.K. Siltuximab for multicentric Castleman's disease: a randomised, double-blind, placebo-controlled trial. *The lancet oncology* **2014**, *15*, 966-974.
48. Younes, A.; Gopal, A.K.; Smith, S.E.; Ansell, S.M.; Rosenblatt, J.D.; Savage, K.J.; Ramchandren, R.; Bartlett, N.L.; Cheson, B.D.; De Vos, S. Results of a pivotal phase II study of brentuximab vedotin for patients with relapsed or refractory Hodgkin's lymphoma. *Journal of clinical oncology* **2012**, *30*, 2183.
49. McGonigle, K.F.; Muntz, H.G.; Vuky, J.; Paley, P.J.; Veljovich, D.S.; Greer, B.E.; Goff, B.A.; Gray, H.J.; Malpass, T.W. Combined weekly topotecan and biweekly bevacizumab in women with platinum-resistant ovarian, peritoneal, or fallopian tube cancer: results of a phase 2 study. *Cancer* **2011**, *117*, 3731-3740.
50. Drilon, A.E.; Camidge, D.R.; Ou, S.-H.I.; Clark, J.W.; Socinski, M.A.; Weiss, J.; Riely, G.J.; Winter, M.; Wang, S.C.; Monti, K. Efficacy and safety of crizotinib in patients (pts) with advanced MET exon 14-altered non-small cell lung cancer (NSCLC). **2016**.
51. Pietrantonio, F.; Morano, F.; Corallo, S.; Miceli, R.; Lonardi, S.; Raimondi, A.; Cremolini, C.; Rimassa, L.; Bergamo, F.; Sartore-Bianchi, A. Maintenance therapy with panitumumab alone vs panitumumab plus fluorouracil-leucovorin in patients with RAS wild-type metastatic colorectal cancer: a phase 2 randomized clinical trial. *JAMA oncology* **2019**, *5*, 1268-1275.
52. Yaeger, R.D.; Cercek, A.; O'Reilly, E.M.; Reidy, D.L.; Kemeny, N.E.; Wolinsky, T.; Gollub, M.J.; Lacouture, M.E.; Rosen, N.; Vakiani, E. Pilot study of vemurafenib and panitumumab combination therapy in patients with BRAF V600E mutated metastatic colorectal cancer. **2015**.

53. Comte, A.; Jdid, W.; Guilhaume, M.; Kriegel, I.; Piperno-Neumann, S.; Dieras, V.; Dorval, T.; Pierga, J.; Cottu, P.; Mignot, L. Survival of breast cancer patients with meningeal carcinomatosis treated by intrathecal thiotepa. *Journal of neuro-oncology* **2013**, *115*, 445-452.
54. Le, D.T.; Uram, J.N.; Wang, H.; Bartlett, B.R.; Kemberling, H.; Eyring, A.D.; Skora, A.D.; Luber, B.S.; Azad, N.S.; Laheru, D. PD-1 blockade in tumors with mismatch-repair deficiency. *New England Journal of Medicine* **2015**, *372*, 2509-2520.
